# Epaisseur de l'intima-média carotidienne et facteurs de risque cardio-vasculaires

**DOI:** 10.11604/pamj.2015.21.153.6876

**Published:** 2015-06-24

**Authors:** Nicolas Fanantenana Herinirina, Lova Hasina Ny Ony Narindra Rajaonarison, Andry Roussel Herijoelison, Ahmad Ahmad

**Affiliations:** 1 Service Imagerie Médicale, Centre Hospitalier Universitaire d'Antsiranana, Madagascar; 2 Service Imagerie Médicale, Centre Hospitalier Universitaire Joseph Ravoahangy Andrianavalona, Antananarivo, Madagascar; 3 Service Imagerie Médicale, Centre Hospitalier Universitaire de Toamasina, Madagascar

**Keywords:** Echographie, épaisseur intima-média, facteurs de risque cardio-vasculaires, Echography, intima-media thickness, cardiovascular risk factors

## Abstract

**Introduction:**

L’épaisseur intima-média de la carotide commune mesurée à l’échographie est un marqueur de risque cardio-vasculaire. L'objectif de ce travail est d’établir la corrélation entre l’épaisseur de l'intima-média carotidienne commune et les facteurs de risque cardio-vasculaire chez des sujets asymptomatiques.

**Méthodes:**

Etude transversale descriptive et analytique portant sur 77 sujets de 40 ans et plus chez qui nous avons évalué les facteurs de risque cardio-vasculaire et analysé leur association avec l’épaisseur intima-média carotidienne commune.

**Résultats:**

L’épaisseur intima-média augmentait avec l’âge. Les hommes avaient une épaisseur intima-média plus marquée que les femmes. L'hypertension artérielle, le diabète et la dyslipidémie sont corrélés à l’épaisseur de l'intima-média contrairement au tabagisme.

**Conclusion:**

L’âge élevé et le sexe masculin sont les facteurs déterminants de la majoration de l’épaisseur intima-média carotidienne commune surtout si s'ajoutent l'hypertension artérielle, le diabète ou la dyslipidémie.

## Introduction

Les maladies cardio-vasculaires et cérébro-vasculaires représentent une cause majeure de morbidité et de mortalité. La mesure de l’épaisseur intima-média (EIM) carotidienne à l’échographie a été proposée afin de mieux préciser le risque vasculaire [1, 2]. Elle constitue un instrument de recherche bien validé qui prend une place de plus en plus importante en pratique clinique [3, 4]. Cette étude avait pour objectif d’établir une corrélation entre l'EIM carotidienne chez des patients asymptomatiques et les facteurs de risque vasculaire (FDR) présentés par ces patients.

## Méthodes

Une étude prospective transversale descriptive et analytique, basée sur les résultats d'exploration échographique et les données enregistrées lors de l'interrogatoire, a été menée au service d'Imagerie Médicale du CHU Ravoahangy Andrianavalona à Antananarivo, Madagascar du 15 mai au 15 aout 2012. Les sujets étaient des patients tous venants référés pour examen échographique et ayant accepté d’être examinés selon le protocole d’étude après une explication du déroulement et de l'objectif de l'exploration. Les patients âgés de plus de 40 ans, sans signe d'accident vasculaire cérébral récent ou ancien ont été inclus dans cette étude. Les sujets chez qui la bifurcation carotidienne était haut située, ont été exclus de l’étude du faite de la difficulté d'exploration de la paroi postérieure du segment distal de la carotide commune occasionnée par ces cas. L'EIM carotidienne était mesurée chez le patient en décubitus dorsal, la tête dans l'axe du corps, à l'aide d'un appareil d’échodoppler vasculaire de marque Mindray DC 6, avec une sonde linéaire de haute fréquence (7,5 MHz). Une coupe longitudinale en mode 2D de l'artère carotide commune était réalisée à 1 à 2 cm en amont de la bifurcation carotidienne. La mise en évidence parfaite des parois superficielle et profonde permettait d'identifier le plus grand diamètre vasculaire. La mesure était réalisée par méthode manuelle (mesure non informatisée) au niveau de la paroi postérieure des carotides communes droite et gauche. Les FDR cardio-vasculaires étudiés et rapportés à l'EIM carotidienne étaient le genre, l’âge, l'indice de masse corporelle (IMC), l'HTA, le diabète, la dyslipidémie portant sur l’élévation du cholestérol total ou du LDL ou diminution du HDL et le tabagisme. Les données ont été traitées sur Microsoft Excel 2007 et analysées sur Epi-Info 3.5.3. Le test est statistiquement significatif si P < 0,05.

## Résultats

**Figure 1:**
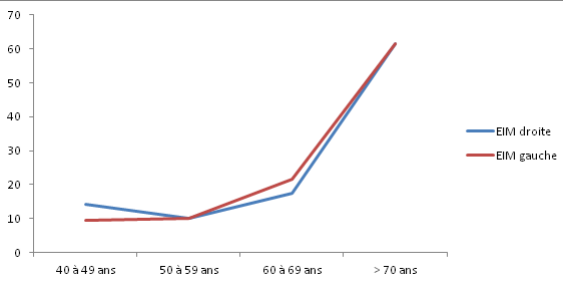
Courbe d’évolution de l’épaisseur intima-média carotidienne en fonction de l’âge

Nous avons retenu 77 patients, âgés de 40 à 79 ans avec une moyenne d’âge de 58,16 ans. L'EIM était plus élevée chez les patients plus âgés (P < 0,05). Elle augmentait de façon significative à partir de 50 ans (Figure 1). Moins de femmes (20,5%) que d'hommes (36,84%) présentaient une EIM augmentée (P = 0,008) (Tableau 1). La prévalence de l'EIM augmentée était nettement élevée chez les sujets hypertendus (P= 0,0034 à droite et 0,0091 à gauche), chez les sujets dyslipidémiques (P = 0,0004) et chez les patients diabétiques (P = 0,01). Il existait une corrélation positive entre l'EIM et l'IMC (P = 0,002). Il n'y avait pas de corrélation entre l'EIM et le tabagisme (P = 0,36). L'EIM moyenne à gauche était plus élevée que l'EIM moyenne à droite. Après croisement, elle était statistiquement significative (P = 9,53 10-^13^).

**Tableau 1:** Epaisseur intima-média carotidienne augmentée en fonction du sexe

EIM > 0,8mm	Femme n (%)	Homme n (%)	P-Value
**Droite**	8 (20,5)	14 (36,84)	0,008
**Gauche**	8 (20,5)	14 (36,84)	0,008

## Discussion

La mesure de l'EIM de l'artère carotide par échodoppler est une méthode simple et non invasive permettant une évaluation précise, à un stade précoce de l'athérosclérose. Depuis la description échographique de la carotide à partir des données histologiques par Pignoli [5], de nombreuses études ont tenté d’établir la relation entre l'EIM et certains FDR cardio-vasculaire. Par défaut de méthode automatique qui offre plus de facilité et de précision [6], nous avons réalisé une mesure manuelle de l'EIM de la carotide commune. Malgré cette limite, ce travail a permis d’étudier les sujets à risque cardio-vasculaire dans la mesure où l'augmentation de l'EIM de la carotide est reconnue comme étant associée à un risque plus élevé de survenue d’événements cardio-vasculaires chez les patients présentant des FDR d'athérosclérose [7].

L´âge est la principale variable liée à l´épaississement carotidien dans tous les segments, à la fois chez l'homme que chez la femme, et dans la population générale [8]. L'EIM augmente de près de 3 fois entre 20 et 90 ans [9]. Certaines études post-mortem montrent que cette augmentation liée à l’âge résulte essentiellement d'un épaississement de l'intima [10]. Cazaubon [11] retrouvait une augmentation de l'EIM chez 45% des sujets asymptomatiques âgés en moyenne de 59 ans. Dans notre étude, l’épaississement est significatif à partir de 50 ans. Toutefois, les normes de l'EIM carotidienne varient en fonction de l’âge et les seuils utilisés diffèrent selon les études [12]. Ces variations sont probablement en rapport avec les techniques utilisées mais aussi à d'autres facteurs notamment ethniques. Ainsi, Chow [13] retrouvaient une EIM carotidienne plus élevée chez les Indiens que chez les Australiens. Dans la Rotterdam Study [14], la valeur moyenne de l'EIM carotidienne était de 0,80 ± 0,16 mm au sein d'une population âgée en moyenne de 70 ans et de 0,76 ± 0,16 mm selon Poncelet [15]. Elle était de 0,63 ± 0,16 mm dans l’étude ARIC chez des patients âgés en moyenne de 54 ans [8]. L'EIM carotidienne moyenne de notre étude, mesurant 0,77 ± 0,21 mm pour une moyenne d’âge de 58,16 ans, est comparable avec les données de la littérature.

Comme dans d´autres études [16], le genre a également été associé à l´EIM carotidienne. En effet, nos résultats ont révélé que la prévalence de l'EIM augmentée est élevée chez l'homme que chez la femme. D'autres travaux similaires ont également montré que l'EIM, chez des sujets présentant des FDR vasculaires, était généralement plus importante chez l'homme que chez la femme, souvent dans la tranche d’âge de 60 à 70 ans [17]. Chez les sujets ne présentant aucun FDR vasculaire, l’épaississement de l'intima-média est plus marqué à partir de l´âge de 40 ans chez l'homme et 50 ans chez la femme [8]. Dans notre étude comme dans la littérature, il existe une corrélation positive entre la valeur de l'EIM carotidienne et de l'HTA. De nombreuses études montraient que les sujets hypertendus ont une EIM carotidienne supérieure à celle des sujets normotendus, particulièrement en cas d´hypertension artérielle systolique [18]. Le contrôle de l´hypertension peut conduire à une diminution des valeurs de l´EIM, comme cela a été révélé par des études longitudinales [19].

Dans notre étude, la prévalence de l'EIM carotidienne augmentée chez les patients dyslipidémiques atteint 44,4% contre 10% chez les sujets non dyslipidémiques (P = 0,0004). Ainsi, dans une étude finlandaise comptant 1224 sujets, il a été démontré que l´épaississement de l´EIM carotidienne est étroitement corrélé au taux de LDL-cholestérol [20]. Ceci a été confirmé par une étude cas-témoins [21]. Signorelli [22] rapportaient une corrélation positive de l'EIM carotidienne avec le cholestérol total (P = 0,0001) comme a confirmé Chow [13] ainsi qu'avec le LDL-cholestérol (P = 0,0001). Parmi nos 14 sujets diabétiques, 43,8% ont eu une intima-média carotidienne épaissie. Chez les patients diabétiques, l'EIM est épaissie par rapport aux sujets non diabétiques quel que soit le sexe et l’âge [23]. Ils ont une incidence deux à quatre fois plus de maladies cardiovasculaires, y compris l'augmentation de l'EIM, par rapport aux personnes non diabétiques notamment de type 2; chez qui aucun FDR a été associé à l’épaississement de l'EIM [24–26].

Dans notre étude, la prévalence des EIM augmentées en fonction de l'IMC est variable. Elle est plus élevée (57,1%) chez les sujets obèses. Ces résultats suggèrent que l´obésité pourrait être un FDR important d´athérosclérose carotidienne. D'après Juo, l´obésité et l´EIM partagent les mêmes facteurs génétiques [27]. La prévalence de l'EIM augmentée (24,1% à droite comme à gauche) chez nos 24 sujets tabagiques n'est pas statistiquement significative (P = 0,36). Par contre, Smilde [28] a réalisé une étude comparative entre 56 sujets non tabagiques et 184 sujets tabagiques sans autres FDR vasculaire, et a conclu que le tabagisme, comme seul FDR vasculaire provoque un épaississement de la paroi des artères carotidiennes communes. Cette différence pourrait être expliquée par la population statistique limitée. Dans notre étude, l'EIM à gauche est significativement plus élevée que l'EIM à droite (P = 9,53 10-13). Cette différence a été décrite par Lemne en 1995 [29].

## Conclusion

Parmi les FDR vasculaire, l’âge et le genre masculin sont les facteurs déterminants de l’épaisseur l'intima-média. Il en est de même pour l'hypertension artérielle, le diabète et la dyslipidémie. Par contre, le tabagisme n'affecte pas significativement sur l'EIM. Nous proposons une application clinique de la mesure de l'EIM, chez les patients avec FDR, dans le dépistage précoce de l'athérosclérose et le suivi des effets thérapeutiques.
